# Event and Comment

**Published:** 1915-05

**Authors:** 


### DENTAL REGISTER

Vol. LXIX MAY 1915 No. 5

### EVENT AND COMMENT.

NEW REGISTRATION LAWS. New Jersey has the newest registration law which will become effective September 1, 1915. To the present law some new features are added which make this law stronger and probably more efficient. The Registration Board is increased to eight members which ought to make its work more thorough as the amount of work to be done and that usually in limited time does not seem sufficient for examiners to get a very good idea of what all of the candidates may know. The law requires that candidates shall be graduates of schools requiring a preliminary education equivalent to a four-year high school and that the reputability of the college shall be decided by the Board of Examiners. An annual registration with a fee of two dollars is a new feature, and provision for reciprocity registration is also provided. A fee of fifty dollars is exacted for reciprocal registration. We are wondering why this fee is so large, it seems out of proportion to the work required for such registrations. The law also makes it practically impossible for advertising associations to use unregistered or untrained help in any branch of the operation service. In general the law follows the trend of the more recent legislation in other states. The enactment and enforcement of such laws can not but help on the cause of higher dental education as they compel colleges to better prepare their men for meeting the increased standards of the Examiners. Our schools must matriculate a better educated class of students, and then give them thorough instruction if they are to keep up with these ever increasing standards. All of which promises to lift standards everywhere.

---

WHAT SHALL WE DO WITH THE DENTAL PULP? Years ago we were taught to preserve the vitality of even a remnant of the pulp if possible. Then when the local anesthetic made it so easy to painlessly remove the pulp and fill the canal we went so far as to destroy pulps that were not even deprived of their natural protection of healthy dentine. Still more recently our crown and bridge workers demanded the removal of pulps of absolutely sound teeth when they were to be crowned or used as bridge supports. We are now confronted with the development of the fact that a serious menace to general health is due to alveolar infections from incompletely filled root canals, and the medical profession is censuring us severely and causing wholesale extractions of all pulpless teeth. Our editors and teachers are asking in great earnestness what we are going to do about it. In the first place we must not get panicky; we have not yet had reliable evidence produced to show the theories advanced are facts. We can do these operations more carefully, with full regard to aseptic precautions. We can revise our systems of crown and bridge work and even cavity preparations to avoid the necessity for root canal surgery. We personally have never been able to see the necessity for devitalizing pulps in sound teeth or other teeth for that matter, in which the pulp is but little liable to die under fillings or crowns that are carefully adjusted. But as a last resort we can, with our X-ray, determine the extent of infection about a root end, and if it is of consequence, with our present means of anesthetising the entire dental structures with safety, we can by surgical procedures excise root ends that become troublesome.

